# Applying a Digital Health Checklist and Readability Tools to Improve Informed Consent for Digital Health Research

**DOI:** 10.3389/fdgth.2021.690901

**Published:** 2021-07-15

**Authors:** Camille Nebeker, Maryam Gholami, Daniah Kareem, Emily Kim

**Affiliations:** ^1^Research Center for Optimal Digital Ethics in Health, Herbert Wertheim School of Public Health and Human Longevity Science, University of California, San Diego, La Jolla, CA, United States; ^2^Design Lab, University of California, San Diego, La Jolla, CA, United States

**Keywords:** informed consent, digital health, internal review board, human factors, human centered design, meaningful consent, Digital Health Checklist tool

## Abstract

**Background:** As research involving human participants increasingly occurs with the aid of digital tools (e.g., mobile apps, wearable and remote pervasive sensors), the consent content and delivery process is changing. Informed consent documents to participate in research are lengthy and difficult for prospective participants to read and understand. As the consent communication will need to include concepts and procedures unique to digital health research, making that information accessible and meaningful to the prospective participant is critical for consent to be informed. This paper describes a methodology that researchers can apply when developing a consent communication for digital health research.

**Methods:** A consent document approved by a US institutional review board was deconstructed into segments that aligned with federal requirements for informed consent. Three researchers independently revised each segment of text with a goal of achieving a readability score between a 6–8th grade level. The team then consulted with an external readability expert well-versed in revising informed consent documents into “plain language.” The resulting text was evaluated using Microsoft Word and Online-Utility accessibility software. The final step involved adding visual images and graphics to complement the text. The Digital Health Checklist consent prototype builder was then used to identify areas where the consent content could be expanded to address four key domains of Access and Usability, Privacy, Risks and Benefits, and Data Management.

**Results:** The approved consent was evaluated at a 12.6 grade reading level, whereas the revised language by our study team received 12.4, 12, and 12.58, respectively. The final consent document synthesized the most readable of the three revised versions and was further revised to include language recommended by the software tool for improving readability, which resulted in a final revised consent readability score of a 9.2 grade level. Moreover, word count was reduced from 6,424 in the original consent to 679 in the rewritten consent form.

**Conclusion:** Utilizing an iterative process to design an accessible informed consent document is a first step in achieving meaningful consent to participate in digital health research. This paper describes how a consent form approved by an institutional review board can be made more accessible to a prospective research participant by improving the document readability score, reducing the word count and assessing alignment with the Digital Health Checklist.

## Introduction

In biomedical and behavioral research conducted by regulated entities, obtaining the prospective informed consent of those who become participants in research a cornerstone of ethical research. The purpose of informed consent is to provide people who are considering whether to participate in research the information necessary to determine if they want to volunteer (1). The regulations along with principles described in the Belmont Report intended to guide ethical research are used to determine what information is typically presented in the consent document (1, 2). The US Federal Regulation for Human Research Protections (see 45 CFR 46.116) lists eight key areas that must be described within the consent form (i.e., purpose, experimental aspects, risks, benefits, etc.). In addition to content requirements, there are guidelines suggesting that consent language be accessible aiming for a 6–8th grade reading level and presented in a setting whereby the individual is able to consider the information without undue influence that may compromise their ability to volunteer.

While informed consent is a demonstration of the ethical principle of “Respect for Persons,” in reality, the practice of composing, delivering and obtaining consent to participate in research is far from perfect. Some of the problems stem from assumptions we, as researchers, make as we engage in what is typically a transactional conversation with a prospective research participant. This dialogue begins with the researcher stating they are conducting research to answer an important question followed by details about who is eligible, what's involved, how data will be collected and so forth. The first problem, which is not trivial, is the assumption that people understand what the scientific method involves; however, many don't and, subsequently misunderstandings follow (3). For example, researchers found that even when people can explain what a study involves, they may experience therapeutic misconception, and believe they will receive a medical care (4). In addition to barriers due to consent content, how it's delivered can also presented challenges for achieving informed consent. A number of studies have looked at steps to improving the consent process for example, among older adults (5), with cognitively impaired individuals (6) and adolescents (7). Yet, as we venture into the digital age, more is needed before we can be confident that informed consent is truly informed (8).

As research involving human participants increasingly occurs with the aid of digital tools (e.g., mobile apps, wearable and remote pervasive sensors), the consent content and delivery process is changing. Using digital strategies, researchers can now recruit and enroll upwards of 20,000+ participants rapidly to study various health conditions. The mPower study is one example where Apple Research Kit was used to host a mobile health study focused on Parkinson's disease with a goal of enrolling 20,000 participants using a mobile e-consent process (9). There was no one-on-one conversation between the researcher and prospective participants—all consent information was delivered remotely by placing information and graphics on the prospective participant's smartphone screen. Wearable and home placed sensors are another method used to passively gather a participant's personal health data in their natural environment, unobtrusively. The challenge with studies that take place using social media platforms, mobile apps or other forms of passive, remote study engagement is in how the researcher conveys the complex concepts of digital data collection or technology delivered interventions so that participants actually understand what participation involves. In the digital research environment, not only is explaining the concept of research important but, addressing potential technology and data literacy challenges is also important (10, 11). While the literature reflects ongoing persistent challenges with the concept of informed consent, little guidance is available to support those in the digital health research community who are working to fit a square peg (current consent paradigm) into a round hole (emerging digital health modalities).

The intent of this paper is to provide guidance to digital health researchers on how they can improve the informed consent communication specific to digital health research. This paper describes the process of developing an accessible consent communication. To demonstrate the process, we used the IRB-approve informed consent document developed for a study that involves body worn sensors to capture natural behaviors between a mom and baby in the home environment. In addition, a new checklist tool and framework to guide the consent deconstruction and reconstruction process was used. The Digital Health Checklist (DHC) is a decision support tool developed with a goal of supporting digital health researchers to design safe and responsible digital health research studies (12), including the content developed for use in obtaining informed consent.

## Methods

### The DHC Tool

**Figure 1:**
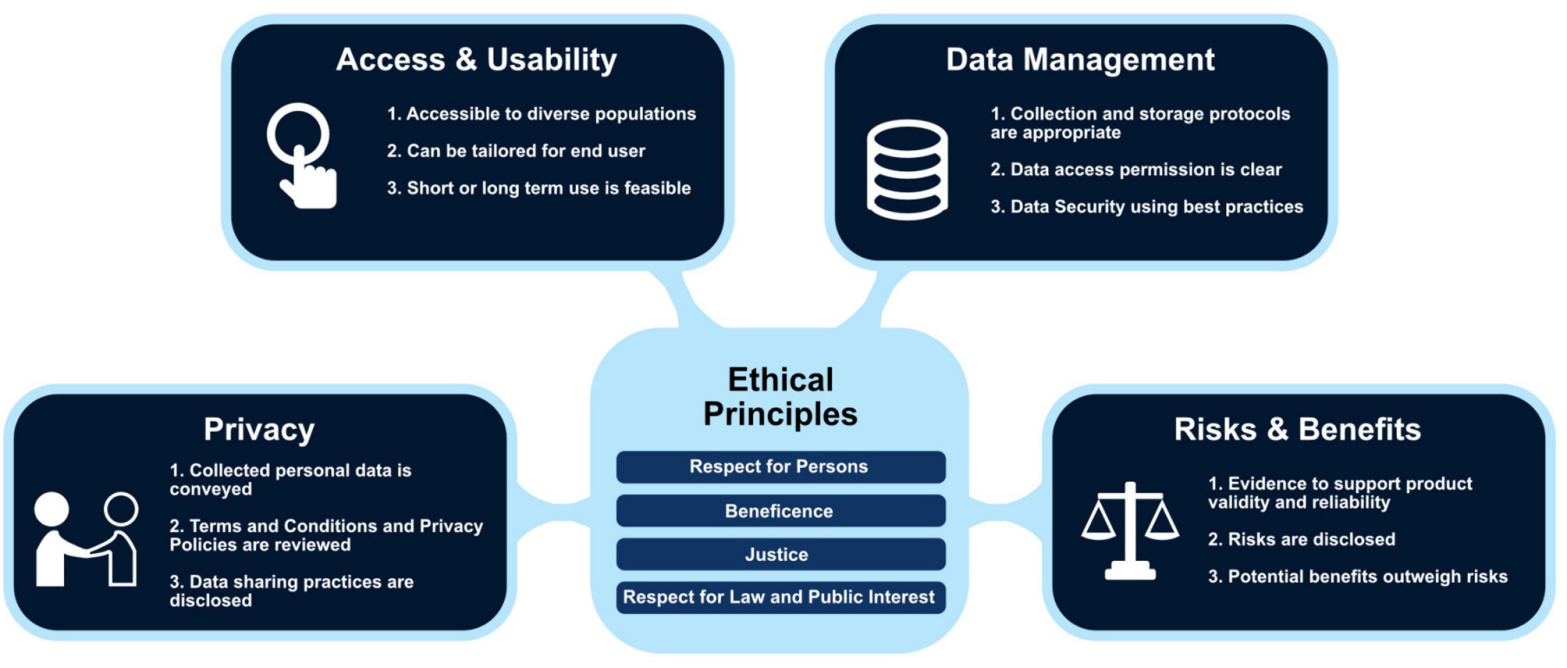
Digital Health Framework with examples of checklist prompts embedded within each domain (Used with permission of C. Nebeker, ReCODE Health).

The DHC was developed via an iterative participatory design process to support decision making during the research protocol and consent development process prior to submission to an ethics review committee [e.g., Institutional Review Board (IRB in the US) or Research Ethics Committee (REC in Canada, European Union)] (12). Given the new challenges in developing ethical digital health studies (e.g., privacy considerations, data management and consent) (see Figure 1), the DHC tool prompts the researcher to consider factors that can influence responsible and safe research practices. The DHC is undergirded by a framework grounded in accepted ethical principles of respect for persons, beneficence and justice (1) augmented by a fourth principle of respect for law and public interest (13). The checklist items are depicted in a matrix table with the vertical listing ethical principles with the horizontal listed the four domains of: Access and Usability, Privacy, Risks and Benefits, and Data Management. For this prototype design process, we used the “respect for persons” section of the DHC as a blueprint to guide the consent content.

### Study Design

This consent design process was initiated to support a longitudinal observational research study that would involve women and children as participants. Specifically, the US National Institutes of Health (NIH) funded a network of researchers to plan the HEALthy Brain and Child Development (HBCD) Study^1^. HBCD will examine early neurological development after prenatal exposure to maternal substance use using a variety of measures, including wearable and remote sensor technologies. The first author was part of an ethics and law working group involved with identifying and examining the ethical, legal and social implications associated with this potentially controversial research. Two passive sensor technologies were being considered for use during the study planning phase. One was a remote sensor that would be placed on the legs of a baby crib/bed to gather respiration and heart rate (14). The other, a body placed sensor that would be worn by the mother and baby to passively collect interpersonal data for specified periods in the home environment. Of the two devices, we selected the IRB-approved consent document developed for the wearable sensor since it involved prospective data collection from research participants.

This study involved a 4-step iterative human-centered design (HCD) process (15). The 4-step HCD include: (1) *Understand and Address the Core Problems*, to solve the fundamental issues, not the symptoms. (2) *Be People-Centered*, as opposed to technology-centered, ensuring that the outcome is appropriate for the culture and environment. (3) *Use an Activity-Centered Systems Approach*, focus upon the entire activity under consideration. (4) *Rapid Iterations of Prototyping and Testing*, and then refine and enhance the capabilities through successive iterations. While application of the HCD process is novel for developing a more accessible consent, we found it useful in conveying our process of developing an accessible consent communication.

#### Understand and Address the Core Problems

The content of informed consent communications used in regulated research is dictated by the federal regulations specific to human subject protections and local Institutional Review Boards. These documents include basic information that an individual may use to decide whether to participate in a research study. Consent communications have become increasingly transactional and include legal disclaimers on top of the basic information about research study participation (e.g., purpose, procedures, risks, benefits, data management, conflicts of interest). This has added to the length of consent communications and has elevated the reading level to around a 10–12th grade, making it difficult for many readers to comprehend (16, 17). To understand a consent communication for digital health research, there are added complexities in that the reader will need, in addition to a level of research literacy, a level of technology and data literacy (18).

The first step was to deconstruct the IRB-approved consent content by breaking it into segments that aligned with the federal requirements elements of informed consent to participate in research (see 46 CFR 46.116), (see Table 1). By doing this, we could focus on what communication was needed to comply with the federal regulations. Upon completion of this step, the research team discussed the challenges they faced while reading each segment and commented on the document length, technical language, and redundant information.

**Table 1:** US federal regulations state that the following information be conveyed to prospective research participants prior to enrolling as a volunteer in research.

**Statement that describes:**	**Detail needed**
• Research study involvement	Explain the purpose, expected duration of participation, what procedures will be followed and a description of experimental aspects.
• Study benefits	Describe any direct benefits to the participant or others, which may be anticipated.
• Study risks	Describe possible risks of harm to the participant.
• Appropriate alternatives	Disclose other options that may be advantageous specific to procedures or possible treatments.
• Confidentiality practices	State how records identifying the participant will be maintained.
• Whether/how injury will be compensated (only if study exceeds minimal risk of harm)	Explain whether compensation is available to cover study related for medical treatment or other injury.
• Study team contact	Identify who to contact if there are questions or to report a research related injury.
• Voluntary nature of participation	Make clear that participation is voluntary and that there is no penalty or loss of benefits if the individual chooses not to participate or changes their mind after initial agreement to enroll.

#### Be People-Centered

Unfortunately, in academic research, researchers are torn between making the consent accessible to those who may be recruited to participate in the research and adhering to the consent template that the IRB wants researchers to follow. The IRB-approved consent for selected for our use-case exceeded the recommended 6–8th grade reading level that IRB guidance suggests. As such, the next step involved our three researchers (MG, DK, EK) independently revised each segment of text with a goal of achieving a readability score of a 6–8th grade reading level. This participatory design process provides valuable insights as the researchers are engaged directly in the task of trying to develop consent language as a researcher would when applying our method to their consent communication process (19).

As noted, a norm of US human research ethics is to aim for a readability score that a majority of the adult population would be able to read, however, rarely is this goal achieved. A challenge was encountered by our team when attempting to revise language occurred when attempting to describe the technology intended for use in the research (passive sensor devices) along with the legal language that the IRB requires. Disclosure of reporting requirements is routine in some studies due to legal requirements like reporting mandates (e.g., disclosure of illegal behaviors like child or elder abuse). That was true for the consent serving as our use-case. The language required by the IRB to convey indemnification and mandated reporting was nearly impossible to reduce to an accessible reading level.

#### Use an Activity-Centered Systems Approach

Once each team member had revised the consent segment to the best of their ability, they reviewed all revisions to ensure alignment with the federal regulations and applied a readability software to assess grade level. The revised segments were analyzed using a readability feature in Microsoft Word, since that tool was compatible for analyzing smaller text segments and accessible to the team. The team members then compared the different versions of text and chose the version that achieved the lowest grade level.

Further iterations were needed to reach the 6–8th grade reading level. The team then consulted with an external readability expert well-versed in revising informed consent documents into “plain language” (20). The external consultant used Readability Studio 1.1 to assess the IRB-approved consent form, which provides grade and difficulty level along with suggestions for how to further simplify the language. The team implemented the suggested wording and finalized the revised document. The final step involved inserting visual images of the technology and graphics to complement the text and improve readability.

#### Rapid Iterations of Prototyping and Testing

The last step involved applying the Digital Health Checklist (DHC) consent prototype tool to identify areas where the consent content could be expanded to address the four domains of Access and Usability, Privacy, Risks and Benefits, and Data Management. Each of the four domains are expanded in the “respect for persons” row of the checklist matrix, which corresponds to what a researcher should consider when developing the informed consent document so that specific information about a digital health strategy/tool can be addressed. Not all of the checklist prompts will be relevant but, the checklist facilitates reflection of what might have been overlooked—particularly if relying on an IRB consent template to guide content. Our team compared what was in the IRB-approved and subsequently revised consent form to the DHC and identified content areas that would need to be added to improve the consent for use in this digital health study.

## Results

### Readability and Content

The original IRB-approved consent form and the revised text were analyzed internally using both Microsoft Word and Online-Utility 1.1, which provide average readability and grade level scores between the two software (see Table 2). The results of the IRB-approved consent showed a 12.6 reading level, whereas the revised language by our study team received 12.4, 12, and 12.58, respectively. The final consent document synthesized the most readable of the three revised versions. We then incorporated language recommended by the software tool for improving readability, which resulted in a final revised consent readability score of a 9.2 grade level. Table 2. Readability analyses and word count of original IRB-approved consent form and final revised version.

**Table 2:** Readability analysis.

	**Original IRB approved consent**	**Rewritten consent by research team**
Word count	2,464	679
Readability grade	Microsoft Word: 13.3 Online-Utility: 11.91	Microsoft Word: 9.3 Online-Utility: 9.13

**Figure 2:**
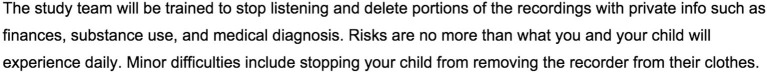
Example of a paragraph in the rewritten version consent form.

**Figure 3:**
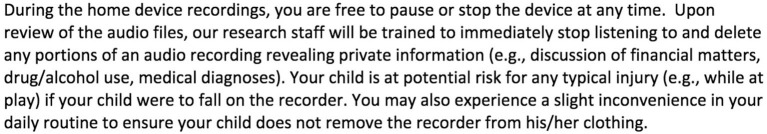
Example of a paragraph in the original IRB-approved consent form.

For a more detailed example of how the text was modified to improve readability, see Figures 2, 3 below which illustrate examples of a paragraph in the rewritten (Figure 2) vs. the original (Figure 3) consent form. This text focuses on risk management and how the study team will be trained to respect participant privacy. The original text was 100 words, and the sentences were much longer when compared to the revised version by 52 words.

The research team was able to improve the readability and lower the reading level of the passages by 3 grade-levels from the original version, however, did not achieve the targeted 6–8th grade reading level. This was achieved by following the plain language guidelines published by the US government^2^, which includes using words with fewer syllables, shorter sentences and shorter paragraphs.

### Presentation and Visuals

**Figure 4:**
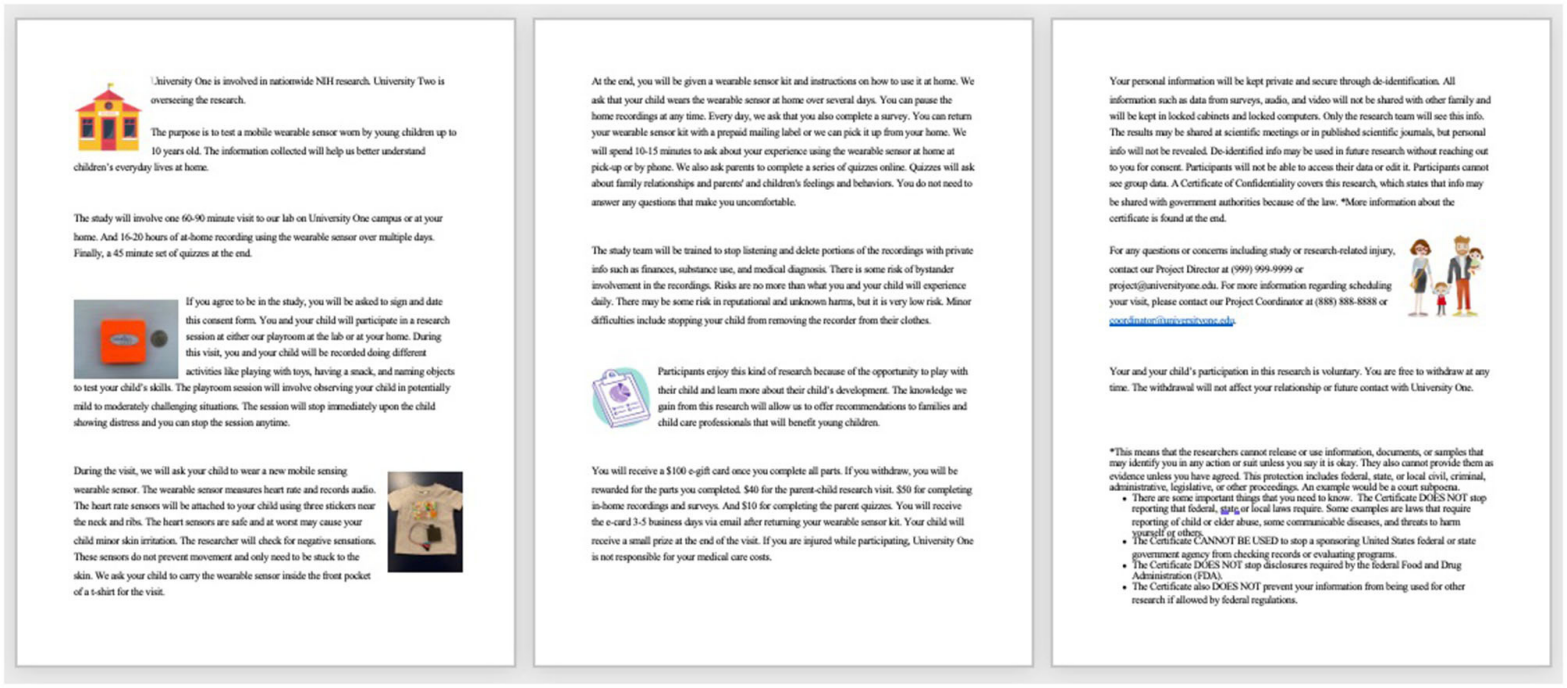
Rewritten version of consent form by research team that includes all required elements of informed consent.

**Figure 5:**
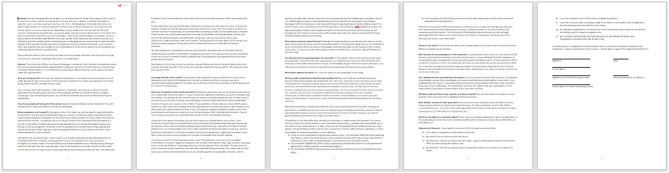
Original IRB approved consent form approved by an IRB.

The final revised version of the consent was augmented to include graphics and pictures of the digital tool. See Figures 4, 5 to compare presentation and visuals of the revised (Figure 4) and original (Figure 5) versions of the consent forms.

The DHC tool was then referenced to identify consent content that could be expanded to address areas specific to Access and Usability, Privacy, Risks and Benefits, and Data Management in the revised consent form. Table 3 illustrates the four domains and their prompts that were used to evaluate the consent form. Under the *Access and Usability* domain, all the statements noted in the DHC tool were addressed in both the original and revised consent document. Under the *Risks and Benefits* domain, which covers potential harms and impact with respect to possible benefits, 10 of the 12 statements were addressed. Under the *Privacy* domain, which covers the extent, purpose, and sharing of personal data, two of the seven statements were addressed in the original consent and carried forward to the revised consent. Under the *Data Management* domain, two of the 10 checklist prompts were addressed.

**Table 3:** Digital health checklist consent blueprint: ethical principle “respect for persons” across four domains.

**Four domains of DHC tool**	**Prompts for each domain**	**Yes**	**No**	**N/A**
Access and usability	1. An explanation about the technology used in the study that helps one to understand: What it does	X		
	2. An explanation about the technology used in the study that helps one to understand: Why it is being used	X		
	3. An explanation about the technology used in the study that helps one to understand: How it works	X		
	4. Plain language is used to describe the commercial vendor agreements: Terms of Service			X
	5. Plain language is used to describe the commercial vendor agreements: Privacy policy			X
	6. Relevant definitions provided using plain language	X		
	7. Access to visual and audio versions of information, if these alternatives are available	X		
Risks and benefits	1. A description of the type of potential harm including: Physical harm (e.g., skin irritation)	X		
	2. A description of the type of potential harm including: Psychological harm (e.g., distress)	X		
	3. A description of the type of potential harm including: 4. Economic harm (e.g., cost that the participants might incur as a result of using the technology)		X	
	5. A description of the type of potential harm including: Unknown harm (Even when these harms remain unknown - a statement acknowledging that there might be harms that are unknown included)	X		
	6. For potential harms a description the known or unknown: Severity		X	
	7. For potential harms a description the known or unknown: Duration	X		
	8. For potential harms a description the known or unknown: Intensity	X		
	9. Strategies for minimizing risks	X		
	10. Strategies for managing risks	X		
	11. Statement that indicates: Possible benefits from knowledge gained during the study	X		
	12. Statement that indicates: Benefits that could be derived by the participant related to the technology	X		
Privacy	1. Nature of personal information collected by the technology	X		
	2. Purpose for which personal information is collected by the technology		X	
	3. Extent of personal information collected by the technology (specific/inclusive list of personal information that will be collected by the technology)	X		
	4. How individual-level data will be shared and with whom, and if this might change in the future after the study		X	
	5. Whether personal data entered and stored in the technology will be de-identified		X	
	6. A description of how a 3rd party may access and use participant information collected during study participation (normally found in a privacy policy when using a commercial device)		X	
	7. Notification if there is a possibility of bystander involvement		X	
Data management	1. Practices for: Data collection by the technology	X		
	2. Practices for: Data security of the data that is collected by the technology		X	
	3. Practices for: Data sharing with other stakeholders		X	
	4. Practices for: Data transfer from technology to other storage		X	
	5. Practices for: Data storage of data that is collected by the technology		X	
	6. Information about who will have access to data collected by the technology		X	
	7. Whether the research data are controlled by the research team or a third party	X		
	8. Whether the participant will have access to individual-level data collected via the technology		X	
	9. Whether the participant will be able to edit individual-level data collected via the technology		X	
	10. Whether the participant will have access to group-level data collected via the technology		X	

While the original consent included basic information specific to Access and Usability deemed necessary for informed consent to occur, the other three domains were lacking. The next iteration of the consent form will be revised to include information about reputational and unknown harms as well as specify why personal data are being collected and where data are stored and the extent of 3rd party access. Moreover, the possibility of a bystander being recorded is important to address and was not included in the original consent. Bystanders are not typically considered when consenting a research participant but, is increasingly important given the passive and pervasive nature of sensor technologies. Given the consent used in this exercise described a study that used a wearable microphone, addressing bystander considerations is appropriate. Lastly, information about data practices including data transfer, storage, and sharing along with how much access participants will have to collected data will be included in the next iteration of the consent document. At that point, the consent form will include all recommendations in the DHC informed consent blueprint, be accessible in terms of reading grade level and will advance to the stage of further iterative design with prospective research participants.

## Discussion

The main objective of this paper was to provide a step-by-step description of developing an informed consent communication. Using a participatory design, we included researchers who are involved with creating consent communications but who have little experience. Few would argue that valid consent requires that a person be provided with adequate and relevant information. Yet, the process of developing an informed consent document is typically guided by a template that the research ethics board provides for the purpose of helping a researcher create a document that complies with federal regulations and institutional practices. Unfortunately, the consent templates do not include guidance on how to make the consent language or presentation of information accessible or particularly meaningful.

What might make informed consent meaningful has been a subject of study though, whether it can be achieved in practice is uncertain. Dranseika et al. (21) suggested that researchers take the time to learn what information might be relevant for prospective participants and actually speak with patients to learn what might contribute to their decisions about participation in a study. Moreover, they called for empirical research to understand the concept of relevance and how consent content might vary depending on socio-economic and cultural background (21). Most empirical research on informed consent to date has focused on comprehension of the consent content and, subsequent understanding of the research. For example (22), designed and tested an instrument to assess participant objective and subjective understanding of a cancer clinical trial (22). Wilbanks (11) recognized that problems may exist in the traditional consent process and explored how the concept of a choice architecture (23) might be used in guiding decision to participate in digital research whereby consent information was presented on a personal mobile device (11). In fact, Wilbanks argued that in an era of technology mediated clinical and biomedical research with the associated volume, velocity and variety of data, that bioethics must meet the new demands. Experimenting with new design elements with a focus on linear, graphic/pictorial and brief narrative, the team at Sage Bionetworks created a consent flow that was used to communicate informed consent content via an iPhone. Similar to other studies, the need to engage people prospectively in the design process was a limitation. Formative research with mPower study participants conducted by (10) similar inconsistencies in understanding as would be found in traditional face to face consent but, highlighted a desire by participants to be partners in research (10).

The importance of engaging “end users,” in this case researchers and, eventually research participants, early in the design phase of a consent design process cannot be understated. Applying human centered design principles to the concept of informed consent makes sense yet, there is limited literature on this topic. The exceptions are the work of (24) who published a conceptual model of design principles for informed consent related to cookie technology and web browser design (24) and Wilbank's work mentioned previously (11). Recognizing the need to move toward a meaningful and accessible informed consent communication for digital health research is what led to the design process described in this paper.

In this study, we have taken steps to bridge the gap in accessible and meaningful informed consent by moving beyond a transactional form to a presentation of information that is likely to be read and understood. An iterative process was used to create consent information that can be presented to a prospective research participant by first improving the document readability score and then aligning content with the Digital Health Checklist tool. By utilizing the DHC “respect for persons” consent prototype builder, we were able to guide alignment with the four domains of: Access and Useability, Privacy, Risks and Benefits and Data Management.

With this revised consent communication as a starting point, we now plan to engage prospective research participants in iterative consent design workshops to move toward the ideal of meaningful consent. The next phase of this research will involve people who may eventually participate in our larger HBCD study. They will be asked to comment on the relevance and clarity of the consent language. Building on the Digital Health Checklist and emerging work on participant-centered and dynamic consent models, we will include prompts for participants to rate the relevance of aspects of digital health research that are unique and challenging.

For this initial work, our goal is to help researcher understand and apply a process for conveying complex topics, via a consent communication using tools to make language accessible and content complete. Areas of interest expressed by researchers, which led, in part, to development of the DHC tool, are framed as “how might we” questions. Examples follow:

- Improve understanding of how the technology works?
- Convey individual and societal implications of the knowledge gained?
- Communicate how personal health information is transmitted and stored to the cloud?
- Describe differences between real-time data collection?
- Respect preferences for privacy and control of personal information?
- Understand the extent of control participants want with respect to managing data?
- Accurately convey how personalized algorithms work to nudge behavior change?
- Gauge acceptance of health technologies among family, coworkers and friends?

Clearly, informed consent to participate in digital health research has received little attention from a human centered design perspective. With increasing interest from large scale programs, like the All of Us Research Program and Patient Centered Outcomes Research Initiative, to engage with research participants as partners in the learning process, the opportunities are exciting. The ethical principle of “respect for persons” requires that we actually do more than create a transaction to demonstrate compliance between a researcher and participant. To authentically demonstrate “respect for persons” is to co-design the consent content and process to improve capacity among researchers so that the person considering study participation is informed and able to make a decision about whether to volunteer. Through a human centered design process, we can move from a transaction to a meaningful exchange of information that may lead to an informed consent in practice.

Our planned summative research will expand the work reported here. We encourage other researchers to replicate this process when creating their consent communications. While the results will vary since each study is unique in context, we are confident our methods, conveyed via an authentic use case, can serve as a concrete example.

## Limitations

The informed consent prototype design process described here has not involved people external to our research team; however, we have confidence that our team is similar to those who would be eligible to enroll. Specifically, co-authors involved with the deconstruction exercise included two members of our research team (EK, DK) who had no prior experience writing or reviewing informed consent documents and one member (MG) who had limited experience with preparing research protocols for IRB review. The senior author (CN) is a subject matter expert in research ethics and did not participate in the deconstruct/rebuild exercise. While we have taken the preliminary steps to make the IRB-approved consent more accessible via a lower readability score, we have not tested the language or obtained feedback on whether prospective participants find the additional information prompted by the Digital Health Checklist to be relevant or meaningful.

## Conclusion

To achieve responsible digital health requires that we design our studies, to the extent possible, with our research participants and put their interests at the forefront. The wild west of the digital health era allows for exciting innovation and yet, without a purposeful philosophy of “respect for persons” at the core, we as a community of researchers, technologists, clinicians and citizens will make avoidable mistakes. This paper describes the initial steps that researchers can apply for creating an accessible informed consent for use in digital health research. By making information developed for prospective participants accessible, we can then take a human centered approach to learning what is relevant and how best to convey information that matters most to those we will include in future research studies.

## Data Availability Statement

The original contributions presented in the study are included in the article/supplementary material, further inquiries can be directed to the corresponding authors.

## Author Contributions

CN conceptualized the project, led the design of prototype development and application of the Digital Health Checklist, and developed the first draft of the manuscript. DK and EK contributed to the consent analysis process, revisions using the readability, checklist tools and created figures, and tables used in this paper. MG led the co-authors (DK and EK) in deconstructing and revising the consent content and prototype development and contributed to the methods and result sections of the manuscript. All authors contributed to the article and approved the submitted version.

## Conflict of Interest

The authors declare that the research was conducted in the absence of any commercial or financial relationships that could be construed as a potential conflict of interest.

## Footnotes

^1^ https://heal.nih.gov/research/infants-and-children/healthy-brain

^2^ https://www.plainlanguage.gov/media/FederalPLGuidelines.pdf
